# Impact of Mental Health on Wellness in Adult Workers

**DOI:** 10.3389/fpubh.2021.743344

**Published:** 2021-12-16

**Authors:** Won Ju Hwang, Hyun Hee Jo

**Affiliations:** ^1^College of Nursing Science, East-West Nursing Research Institute, Kyung Hee University, Seoul, South Korea; ^2^Department of Nursing, Hyejeon College, Hongseong, South Korea

**Keywords:** mental health, stress, depression, anxiety, wellness

## Abstract

Wellness in adult workers is intimately associated with better quality of life in individuals, as well as in the family, community, workplace, and country. This study aimed to examine the status of wellness in adult workers and to identify the factors that influence wellness. A descriptive survey was conducted with 260 adult workers. T- test and one-way ANOVA with *post-hoc* Scheffe test were used to analyze the data. Multiple regression analysis was performed on stress, depression, anxiety, well-being, self-efficacy, and perceived health status. The regression model for wellness in adult workers was significant (*F* = 42.21, *p* < 0.001), with an explanatory power of 0.558. Depression (β = −0.306) was identified as the most influential factor for wellness in adult workers, followed by self-efficacy (β = 0.280) and well-being (β = 0.264). Depression, stress, and anxiety negatively influenced wellness, whereas self-efficacy, well-being, and perceived health status positively influenced wellness. Study findings suggest the need to develop intervention programs for workers that decrease stress, depression, and anxiety, and incorporate self-efficacy strategies.

## Introduction

Promoting mental health in adults has great public health and social significance (1). Recently, the COVID-19 pandemic has placed an unprecedented burden on the mental health and wellness of affected workers (2). In the past, the goal of health promotion was focused on physical health; however, today, the idea of health has extended beyond disease prevention to proactive engagement in behaviors that promote optimal health until the last day of life (1, 3). Being in good health can increase happiness and improve quality of life because of related continuous and balanced physical, emotional, social, intellectual, and mental components (4). Optimal health is intimately interrelated with lifestyles and lifestyle habits.

### Assessing Wellness in Adult Workers

This interrelatedness is explained with the concept of wellness. “Wellness” has been defined “as a way of life oriented toward optimal health and well-being in which body, mind, and spirit are integrated by the individual to live more fully” (4–6). Some researchers have referred to wellness as a new concept of health, that is, a new focus on artistic and scientific practices and endeavors that help change lifestyles so that the body and mind are in a state of optimal well-being (3, 4, 7). As shown above, there are several proposed definitions of wellness. According to the World Health Organization (WHO), wellness is “the optimal state of health, that is, the realization of the fullest potential of an individual physically, psychologically, socially, spiritually, and economically, and the fulfillment of one's role expectations in the family, community, place of worship, workplace and other settings.” (8, 9). Although wellness has various definitions, the WHO and many researchers regard it as the realization of one's potential and an active life process toward an optimal state of health (5, 8–10). In the United States, policy based on the concept of wellness was introduced for the first time through the Health People 2000 initiative. Later, its importance was highlighted in the Patient Protection and Affordable Care Act (Obamacare), in which the creation of healthy communities and the implementation and evaluation of workplace wellness programs were explicitly stated in law (9, 11). In Korea, economic growth and increase in per capita income has led to more interest in the health and quality of life of citizens, which have been increasing; the use of the term “wellness” has been spreading rapidly since the 2000s (12). Therefore, understanding how adult workers perceive wellness to achieve their potential and carry out desirable lifestyles is a meaningful research issue (13). McCoy, Stinson (14) found that the wellness program consisted of various topics such as physical fitness, smoking cessation, cancer, risk reduction, cardiovascular disease prevention, violence prevention, and mental health. The mental health of workers is related to their wellness (15). Richardson (16) suggested that the spread of mental health promotion programs and wellness programs aimed at mental health is necessary, and stress management of workers is an effective program for mental health (17). Therefore, it is necessary for the workers' mental health, to examine the effects of stress, depression, anxiety, and self-efficacy that explain mental health on wellness (10, 18).

Most of the previous studies on wellness have been conducted with college students, adults, or older adults, and only a few have examined wellness in adult workers (12, 19, 20). In the 2011 World Economic Forum in Davos-Klosters, Switzerland, workplace wellness was one of the major topics. WHO, also, developed the mental health action plan 2013–2020 [20]. High wellness levels in workers play a key role in improving productivity in the workplace and national well-being (9). In addition, research on wellness promotion has focused primarily on physical or leisure activities (12, 21, 22), and few studies have been conducted on the relationship between mental health and wellness, and more research is needed.

Wellness in adult workers is not only associated with the improvement of quality of life of individuals, but also closely linked to the well-being of the family, community, workplace, and the country. Therefore, it is important to identify the status of wellness in adult workers and investigate the factors influencing their wellness.

The purpose of this study was to identify factors influencing wellness in adult workers and provide the basis for the development of wellness program for workers. Specific study objectives were as follows: First, to investigate difference in the wellness index according to subjects' demographic characteristics. Second, to investigate the level of impact of stress, depression, anxiety, well-being, self-efficacy, and demographic characteristics on the wellness index.

## Methods

### Study Design

A descriptive survey was carried out using a self-report questionnaire, with the aim of identifying the factors that influence the wellness in adult workers. The study was carried out after approval of the institutional review board of K University [KHSIRB-20-192 (EA)].

### Data Selection

The target population was adult workers aged 20 years or older who did a specified type of works in a specified way during the last 7 days in the workplace. Inclusion criteria of study participation was aimed at workers who wished to participate in the research by posting on-line promotions and posters at workplaces (schools, companies, factories, offices, organizations, etc.), and those who agreed to participate in the research by explaining the purpose and method of the research.

Questionnaires were forwarded to subjects who agreed to participate in the study using an online platform survey. Excluded subjects were those who were taking psychiatric drugs, and those who were receiving psychiatric specialized treatment were excluded. All subjects had been made aware of the voluntary nature of their participation. The actual number of participants is unknown because information on subjects who did not respond to the questionnaire after access or were excluded among the study subjects is unknown. The final questionnaire was answered by 262 subjects. In 262 subjects, outlier test was performed, and two subjects with a residual of ±2 or more were found and deleted. Regression analysis was performed in 260 subjects.

### Instruments

#### Stress

Stress was measured with the Korean version of the Perceived Stress Scale (K-PSS) (23), which was developed by Cohen, Kamarck (24) to measure subjective perception of stress. This scale consists of six items measuring negative perception and four items measuring positive perception. Subjects are instructed to consider the past 1 month when responding to the items. Each item is presented with five answer choices scored from 0 to 4 points. The total score ranges from 0 to 40 points, and the higher the total score, the greater the perceived stress. As for the interpretation of the results of this measurement, the normal state is 13 points or less, mild stress state is 14–16 points, the moderate stress state is 17–18 points, and the severe stress state is 19 points or more. Cronbach's α of K-PSS was 0.82 (23), The value of Cronbach's α was 0.89 in this study.

#### Depression

Depression was measured with the Korean version of the Patient Health Questionnaire-9 (PHQ-9). The Patient Health Questionnaire-9 is a self-report scale developed by Kroenke, Spitzer (25), and the Korean version, which had been used in this study, was translated by Lee, Huh (26). Subjects are instructed to respond by checking the frequency with which they have been bothered by depression symptoms in the past 2 weeks. The total score ranges from 0 to 27 points and the higher the total score, the more severe the depression. The interpretation of the results of the depression score is 0–4 points for normal, 5–9 points for mild depression, 10–19 points for moderate depression, and 20–27 points for severe depression. Cronbach's of Korean version of the Patient Health Questionnaire-9 (PHQ-9) was 0.85, The value of Cronbach's α was 0.87 in this study.

#### Anxiety

Anxiety was measured with the Korean version General Anxiety Disorder-7 (translated by the author and distributed free of charge at www.phqscreeners.com) developed by Spitzer, Kroenke (27). The General Anxiety Disorder (GAD)-7 is also a self-report instrument, and is a diagnostic tool for generalized anxiety disorder based on the GAD diagnostic criteria of the Diagnostic and Statistical Manual of Mental Disorders, Fourth Edition. The total score ranges from 0 to 21 points, and a higher total score indicates a higher level of anxiety. The interpretation of the results of the anxiety score is 0–4 points for normal state, 5–9 points for mild state, 10–14 points for moderate state, and 15 points or more for severe state. At the time of development, Cronbach's α was 0.92 and Cronbach's α in this study was 0.91.

#### Well-Being

Dodge et al. (28) defined well-being as “the balance point between an individual's physical, psychological, and social resource pool and the physical, psychological, and social challenges faced” (28, 29). Well-being was measured with the WHO-5 Well-Being Index (30). The English version of the WHO-5 was translated into Korean by two independent bilingual experts, and the translated one was reverse-translated from the Korean version into English by an expert in English. The WHO-5 consists of five items on well-being (the state in which body and mind are enriched and all is harmonious) in the past 2 weeks. For each item, the response is based on a 6-point scale. The total score ranges from 0 to 25 points and the higher the score, the higher the level of well-being. Cronbach's α was 0.85 in the prior study (31) and the value of Cronbach's α was 0.95 in this study.

#### Self-Efficacy

Self-efficacy was measured with the Korean version of the Self-Efficacy Scale. The scale was originally developed by Gordon et al. [29] and translated into Korean by Hong [30]. It consists of a total of 10 items, each with responses based on a 4-point Likert scale. The higher the score, the higher the self-efficacy. The value of Cronbach's α was 0.88 in this study.

#### Wellness

Wellness refers to continuous pursuit of a positive state (4–6) or an optimal state of health (4) by going beyond mere absence or avoidance of disease and striving for harmonious integration of body, mind, and environment. Wellness was measured with the instrument developed by Wilson and Ciliska (32) and two experts translated into Korean and revised with supplementation. The instrument has a total of 42 items, each with responses based on a 5-point scale. It consists of three subcategories for all regions 12 questions for physical wellness, seven questions for social wellness, three questions for spiritual wellness, nine questions for emotional wellness, five questions for intellectual wellness, and six questions for occupational wellness. Each item is scored between 1 and 5 points, and the higher the score, the higher the wellness index. The value of Cronbach's α was 0.95 in this study.

### Data Analysis

Data analysis was performed using IBM SPSS Statistics v25.0 (IBM Corp., Armonk, NY, 173 USA). “Subjects” demographic characteristics are reported as frequencies and percentages. The comparison of wellness scores according to the general characteristics of the subjects was conducted by *t*-test and one-way ANOVA with *post-hoc* Scheffe test.

The relationships between wellness and various relevant factors (stress, depression, anxiety, well-being, self-efficacy) and multicollinearity were examined by computing Pearson correlation coefficients. In addition, to identify factors influencing wellness in adult workers, multiple regression analysis was performed and the assumptions of the model were then checked. Using G^*^Power, with an effect size of 0.15 (medium), power 0.95, alpha of 0.05 and with five predictor variables, a minimum number of 138 subjects was required for multiple regression (33).

## Results

### Demographic Characteristics

To examine subjects' demographic characteristics, data on gender, age, education level, marital status, monthly income, job type, number of daily working hours, and perceived health status were collected (Table 1). Of all the subjects, 45.4% were men and 54.6% were women. The most common age group was 30–39 years, comprising 53.1% of the study sample. Married subjects comprised 60.8% of the sample. The percentage of subjects who were college graduates or higher was 83.8%. The percentage of subjects with a monthly income of 3 million Korean won or more 71.2%. Office workers comprised 74.6% of the sample. Mean number of daily working hours was 8.67. Lastly, in terms of perceived health status, 45.6% perceived their health to be fair.

**Table 1:** Wellness scores according to the characteristics of the subjects (*N* = 260).

**Variables**	**Category**	***N* (%)**	**Mean ± SD**	***t* or *F***	***p***	**Scheffe**
Gender	Men	118 (45.4)	2.82 ± 0.38	1.155	0.279	
	Women	142 (54.6)	2.76 ± 0.38			
Age	20–29_a_	48 (18.5)	3.07 ± 0.54	2.985	0.020	e < a, b < c, d
	30–39_b_	138 (53.1)	3.14 ± 0.51			
	40–49_c_	54 (20.8)	3.27 ± 0.54			
	50–59_d_	14 (5.4)	3.43 ± 0.62			
	60–69_e_	6 (2.3)	2.66 ± 0.78			
Marital status	Single_a_	95 (36.5)	3.09 ± 0.51	3.256	0.040	a = b = c
	Married_b_	158 (60.8)	3.23 ± 0.55			
	Others_c_	5 (1.9)	2.78 ± 0.78			
Education level	High school diploma or less	39 (15.0)	2.75 ± 0.49	−0.864	0.389	
	College or more	218 (83.8)	2.80 ± 0.35			
Monthly income	<3 million KW	75 (28.8)	2.66 ± 0.44	−3.368	0.001	
	3 million KW or more	185 (71.2)	2.84 ± 0.34			
Type of job	Office workers	194 (74.6)	2.79 ± 0.40	0.923	0.357	
	Factory workers	56 (21.5)	2.74 ± 0.30			
Number of working hours (day)		8.67 ± 2.03				
Perceived health status	Poor_a_	55 (21.2)	2.79 ± 0.55	29.28	<0.001	a < b < c
	Fair_b_	118 (45.6)	3.13 ± 0.46			
	Good_c_	86 (33.2)	3.44 ± 0.51			

*_abcde_ = The Scheffe post-hoc test was performed; KW, Korean won*.

### Wellness-Related Factors

The mean (and standard deviation) wellness was 3.17 ± 0.55, as shown in Table 2. The scores of the factors influencing wellness were as follows: Overall mean value of stress was 17.49 ± 4.10, which was above the normal cut-off score, only 16.2% were in the normal range, and 42.1% were in a severe state. The mean of depression was 6.15 ± 4.72, indicating mild depression, and 43.2% were normal range. The mean of anxiety was 4.15 ± 4.9, which could be interpreted as a normal range, and 62.5% was found to be normal range. Well-being was 7.91 ± 5.28, and self-efficacy was 2.79 ± 0.38.

**Table 2:** Associated factors of wellness and wellness scores (*N* = 260).

**Variable**	**Mean ± SD/*n* (%)**	**Minimum**	**Maximum**
Stress	17.49 ± 4.10	5.00	32.00
Normal ( ≤ 14)	42 (16.2)		
Mild (14–16)	48 (18.5)		
Moderate (17–18)	60 (23.2)		
Severe (≥19)	110 (42.1)		
Depression	6.15 ± 4.72	0.00	27.00
Normal (0–4)	113 (43.2)		
Mild (5–9)	86 (33.2)		
Moderate (10–19)	60 (23.1)		
Severe (20–29)	1 (0.4)		
Anxiety	4.15 ± 4.09	0.00	21.00
Normal (0–4)	163 (62.5)		
Mild (5–9)	63 (24.3)		
Moderate (10–14)	31 (12.0)		
Severe (≥15)	3 (1.2)		
Well-being	7.91 ± 5.28	0.00	25.00
Self-efficacy	2.79 ± 0.38	1.00	4.00
Wellness	3.17 ± 0.55	1.02	4.61

*SD, standard deviation; Sum Mean = Stress, Depression, Anxiety, Well-being*.

### Correlations Between Wellness and Associated Factors

Correlation results are shown in Table 3, with all relationships statistically significant. Negative correlations with wellness were found for stress (*r* = −0.481), depression (*r* = −0.596) and anxiety (*r* = −0.496). As these mental health indicators increase, wellness decreases. Positive correlations were found for well-being (*r* = 0.553) and self-efficacy (*r* = 0.517); as these increase, so too does wellness.

**Table 3:** Correlational analysis of the relationships between wellness and associated factors (*N* = 260).

	**Wellness**	**Stress**	**Depression**	**Anxiety**	**Well-Being**	**Self-Efficacy**
Wellness	1					
Stress	−0.481^**^	1				
Depression	−0.596^**^	0.573^**^	1			
Anxiety	−0.496^**^	0.561^**^	0.832^**^	1		
Well-being	0.553^**^	−0.466^**^	−0.499^**^	−0.460^**^	1	
Self-efficacy	0.517^**^	−0.396^**^	−0.455^**^	−0.478^**^	0.414^**^	1

** *p < 0.01*.

### Factors Influencing Wellness

A multiple regression analysis was conducted on stress, depression, anxiety, well-being, self-efficacy, perceived health status, education level, and gender in order to examine the level of influence of these variables on wellness. The results are presented in Table 4. Education level and perceived health status were transformed to dummy variables prior to conducting the analysis. Tests of the assumptions of regression analysis with respect to the aforementioned findings revealed that all the assumptions were met. The Durbin-Watson test was performed to test for autocorrelation in residuals. The test statistic was 1.988, a value greater than the critical value (1.74), and thus, it was concluded that there was no autocorrelation. Multicollinearity was tested using tolerance and variance inflation factors values. The tolerances were under 0.1, and variance inflation factors values did not exceed 10. Hence, it was concluded that multicollinearity was not an issue. Cook's D was used to determine the presence or absence of influential outliers, and it was found that none out of 260 subjects had a Cook's D value over 1.0. Lastly, the results of residual analysis showed that linearity, normality, and homoscedasticity assumptions were satisfied. The regression model for wellness was statistically significant (*F* = 41.21, *p* < 0.001) and the model's explanatory power was 0.558. The most influential factor for the wellness index in adult workers was depression (β = −0.306), followed by self-efficacy (β = 0.280) and well-being (β = 0.264). The regression model for wellness was as follows.

**Table 4:** Results of the multiple regression analysis of the associated factors of wellness (*N* = 260).

**Variables**	***B***	**SE**	**β**	***t***	***p***	**Adj *R*^2^**	***F***
Constant	1.431	0.292		4.907	<0.001	0.558	41.21^***^
Perceived health status	0.139	0.031	0.210	4.522	<0.001		
Stress	−0.017	0.007	−0.129	−2.350	0.020		
Depression	−0.036	0.009	−0.306	−3.807	0.000		
Anxiety	0.025	0.011	0.181	2.315	0.021		
Well-being	0.028	0.005	0.264	5.208	<0.001		
Self-efficacy	0.404	0.072	0.280	5.645	<0.001		
Gender	0.151	0.047	0.136	3.202	0.002		
Education level	0.162	0.066	0.105	2.443	0.015		

*** *p < 0.001; Durbin-Watson = 1.988; dummy variable: Education (College or more:0), Perceived health status (good:0)*.

## Discussion

This study was conducted with the aim of identifying the mental health factors that influence wellness in adult workers. To do so, the relationships between the wellness index and stress, depression, anxiety, well-being, and self-efficacy were examined and the extent to which each of the factors impacted the wellness index was investigated.

Significant difference in wellness was observed for some demographic characteristics. With regard to age, the wellness index was the highest in the age group of 50–59. For education level, the index was higher in the subjects with college education or higher. Married subjects had the highest wellness index (3.23 ± 0.55) compared with those in other marital status categories. Subjects earning 3 million Korean won or more per month had a higher wellness index (2.84 ± 0.34) than those earning less, and subjects who perceived their health as good showed the highest wellness index (3.44 ± 0.51). These findings are consistent with the findings reported in Ha and Park (20) and Ha and Yang (21).

Overall mean value of the wellness index in adult workers was 3.17 ± 0.55 (range: 1–5), indicating moderate level of wellness. The wellness index reported in Ha and Yang (21) was also at the moderate level (3.45 ± 0.55), similar to the current study. Wellness involves an active striving toward an optimal state of health (5, 8, 9). Thus, a low wellness index in adult workers could be linked to low quality of life and reduced job performance at work at a personal level and to economic loss due to decreased productivity at a macro level (21). Accordingly, it would be important to develop interventions to improve wellness in adult workers suffering from mental health problems such as stress, anxiety, and depression due to excessive workload in the pandemic infectious disease era (34).

Workers' psychological factors such as stress, anxiety and depression can come from daily life as well as the workplace because workers spend most of time in the workplace. As we didn't want to restrict our argument to stress, anxiety and depression due to work-related factors, we chose to explore general stress, even though research has shown that stress and depression can be related to work-based factors (35).

However, as a result of evaluating programs for wellness, the smaller the workplace, the less interest in wellness and less programs, and less ability to implement health promotion (14). In addition, there are research results suggesting that the health promotion support of organizational leaders has a positive relationship to employee participation (15). Therefore, it is necessary to increase the wellness of workers and to lower the barriers to entry of programs for health promotion. Also, it would be of benefit to employees if wellness programs were made available in the workplace.

The regression analysis showed that perceived health status, stress, depression, anxiety, well-being, self-efficacy, and education all affected the wellness index. Of those, stress and depression were significantly negatively correlated with wellness, whereas anxiety, well-being, self-efficacy, and perceived health status were significantly positively correlated with it. That is, wellness improved, as stress and depression decreased and anxiety, well-being, self-efficacy, and perceived health status increased. This finding supports past research indicating that the wellness index has a significant negative correlation with perceived stress (20). It is explained that stress is highly likely to react differentially in various work environments, and the most important thing in predicting and managing health outcomes is perceived stress by individuals (15, 36). Anxiety and wellness were negatively correlated, but in the regression analysis, anxiety was positively associated with wellness after controlling for all the other variables. Most of the studies describe the relationship between anxiety and wellness as a negative relationship. However, this study showed a positive relationship, which is possibly due to anxiety in this study being within the normal range (37).

Of the outcome variables examined in this study, depression had the largest negative impact on wellness. This finding is consistent with a previous study that indicated that depression was a critical variable in explaining quality of life (38). In the present study, the mean depression score was 6.15 ± 4.72 and according to the Patient Health Questionnaire-9 criteria, scores between 5 and 9 indicate mild depression. As the previous studies have shown that depression significantly increases in old age (38, 39), workers in this study could be considered to be in the age groups for which depression is likely to increase in the future because more than 70% of workers are <40 years of the age. Hence, as found in this study, wellness is more negatively influenced as depression increases. In addition, stress was also found to have negative impact on wellness, consistent with the findings of Ha and Park (20).

Other researchers have reported that their subjects were under continuous stress (20, 40). We further decided to employ a general stress scale as previous research in a similar intervention for employees showed that work-related and non-work-related problems are equally often indicated and addressed (40). As discussed above, half the subjects reported severe stress and around 23% reported severe depression, therefore some adult workers in the study suffered from severe stress and depression, which negatively affected their wellness. Tetrick and Winslow (41) found that recent job stress management programs have focused primarily on reducing negative aspects of well-being (e.g., stress). As interest in positive psychology has increased, the effects of positive interventions have been investigated, and the focus is not on mitigating only the negative aspects, but increasing the positive aspects. The benefit of the aspect is that it allows individuals to do their own activities. It means that interventions that focus on enhancing positive aspects by allowing individuals to pursue activities of their own choice have been found to be of benefit (42). These findings suggested that there is an urgent need for mental health promotion programs for adult workers (20).

Results of the current study also indicated that self-efficacy was a positive factor for wellness. In a study conducted by Shim et al. (43), depression was positively correlated with stress (i.e., the higher the depression level, the higher the stress) and negatively correlated with self-efficacy (i.e., the higher the depression, the lower the self-efficacy), suggesting that stress and depression may decrease as self-efficacy increases. This finding is similar to those of Ha and Yang (21), who examined exercise self-efficacy in workers, and of Lee et al. (44) who examined self-efficacy in women college students. According to Bandura (45), individuals with high self-efficacy do not give up and keep striving based on the expectation of the self and self-confidence. Hence, if self-efficacy is increased, individuals are more likely to strive toward wellness even when faced with an extrinsic factor or a situation impeding health promotion or quality of life improvement. In this regard, self-efficacy can improve through the experience of achievements and positive feedback (46). Therefore, developing intervention programs for the improvement of self-efficacy in adult workers is crucial for increasing wellness in this population.

In the current study, perceived health status had positive impact on wellness. Specifically, compared with the subjects who perceived their health as good, those who perceived their health as fair or poor had lower levels of wellness. One previous study demonstrated that the more favorable the perceived health status, the higher the level of health promoting behavior (21). Perceived health is not an objective index but indicates subjectively perceived levels of health (47). The study finding demonstrates that individuals who perceive their health as good are more likely to carry out health promoting behaviors, consequently affecting wellness, and that subjective perception of good health is a critical variable in bringing positive changes in life (48). Wellness was also influenced by well-being. Well-being refers to subjective perception of the extent to which self is in the state of enriched body and mind and harmony (49). It has been reported that individuals who positively perceive their health are more likely to continue making efforts for personal wellness (8, 9). Accordingly, individuals should be encouraged to carry out health promoting behaviors and be steered toward wellness through diverse health management programs that increase health-related awareness.

There are some limitations. The impact of stress, depression, anxiety, perceived health status, well-being, self-efficacy on wellness was investigated for adult workers, particularly their mental health, however we have not distinguished between office and factory workers due to the insufficient information (35). Despite these positive results, as this is a preliminary study, it has some limitations including small sample size and lack of classification of type of workers (2). We suggest an extension of this study with a wider sample and clear classification between white and blue-collar workers. Future studies should incorporate controlling other factors such as work classification and work-related environment, and more factors such as measure of work performance and organizational level should be incorporated in the future research. Also, high job stress due to a high-intensity work-related environment can be problematic in the workplace. Therefore, it is thought that an intervention study that approaches both job stress and depression is needed in the future.

## Conclusion

The purposes of this study were to examine the mental health status of adult workers by assessing stress, depression, anxiety, perceived health status, well-being, self-efficacy, and wellness, and to identify factors influencing wellness. The study results showed that wellness is affected by stress, depression, anxiety, well-being, self-efficacy, and perceived health status. The findings suggest that mental health influences wellness in adult workers and mental health should be improved to increase wellness. They further indicate that there is a need to develop intervention programs that reduce stress, depression, and anxiety, and that methods of enhancing self-efficacy should be incorporated into these programs.

This study had one major limitation. Because the study sample of adult workers was a convenience sample, the findings may not be generalizable to all adult workers. In the future, follow-up research should be conducted with subjects of diverse occupations and various age groups. Additionally, there is a need for mental health promotion programs that promote wellness in adult workers, and studies should be conducted to develop such intervention programs and to test for their effectiveness.

## Data Availability Statement

The raw data supporting the conclusions of this article will be made available by the authors, without undue reservation.

## Ethics Statement

The studies involving human participants were reviewed and approved by Kyung Hee University. The patients/participants provided their written informed consent to participate in this study.

## Author Contributions

WJH and HHJ participated in the design of this study, analyzed the data and interpreted the results, and wrote the manuscript. WJH directed this study. All authors contributed to the article and approved the submitted version.

## Funding

This research was supported by a grant from the Korea Health Technology R&D project through the Korea Health Industry Development Institute (KHIDI), and it was funded by the Ministry of Health & Welfare, Republic of Korea (grant number: HI18C1317). The funding agencies had no role in the study design, the collection, analysis, or interpretation of data, the writing of the report, or the decision to submit the article for publication.

## Conflict of Interest

The authors declare that the research was conducted in the absence of any commercial or financial relationships that could be construed as a potential conflict of interest.

## Publisher's Note

All claims expressed in this article are solely those of the authors and do not necessarily represent those of their affiliated organizations, or those of the publisher, the editors and the reviewers. Any product that may be evaluated in this article, or claim that may be made by its manufacturer, is not guaranteed or endorsed by the publisher.
